# Evaluating ChatGPT-5’s Performance in Answering Common Patient Questions About Femoroacetabular Impingement and Hip Arthroscopy

**DOI:** 10.1007/s43465-026-01696-3

**Published:** 2026-02-02

**Authors:** Maximilian Voss, Hannah Jaeger, Mikhail Salzmann, Robert Prill, Timoty Osterberger, Ingo J. Banke, Nikolai Ramadanov

**Affiliations:** 1 Center of Orthopaedics and Traumatology, Brandenburg Medical School, University Hospital Brandenburg/Havel, Brandenburg an der Havel, Germany; 2 Faculty of Health Science, Brandenburg Medical School, Brandenburg an der Havel, Germany; 3 Clinic of Orthopaedics and Sports Orthopaedics, School of Medicine and Health, TUM University Hospital, Technical University of Munich, Munich, Germany; 4 AGA-Society for Arthroscopy and Joint-Surgery, Hip Committee, c/o Walder Wyss Ltd, Zurich, Switzerland

**Keywords:** Artificial intelligence, Large language models, ChatGPT-5, Hip arthroscopy, Femoroacetabular impingement

## Abstract

**Background:**

Hip arthroscopy (HAS) is widely used to treat femoroacetabular impingement syndrome (FAIS), and many patients rely on online resources for medical information. Large language models (LLMs) such as ChatGPT have shown potential as supplementary educational tools in orthopedics; however, existing evaluations are limited to earlier model generations with variable accuracy and completeness. This study aimed to evaluate the accuracy, clarity, relevance, and completeness of ChatGPT-5 responses to common patient questions regarding FAIS and HAS.

**Methods:**

ChatGPT-5 was used to generate 25 frequently asked patient questions and corresponding answers related to hip preservation. Two fellowship-trained hip preservation surgeons independently evaluated each response using a five-point Likert scale across four predefined domains: relevance, accuracy, clarity, and completeness. Descriptive statistics were calculated as mean ± standard deviation for each domain. Inter-rater reliability was assessed using a two-way random-effects intraclass correlation coefficient with absolute agreement (ICC [2, 1]) and complemented by exact agreement percentages.

**Results:**

All responses received excellent scores, with mean values ranging from 4.84 ± 0.27 (completeness) to 5.00 ± 0.00 (relevance). Accuracy (4.97 ± 0.08) and clarity (4.91 ± 0.17) were near-perfect. ICC values demonstrated moderate to excellent agreement (0.70–0.81), complemented by high exact agreement rates (84–100%). No answer contained factually incorrect, misleading, or unsafe information. Minor reductions in completeness were attributable to occasional brevity rather than substantive omissions.

**Conclusion:**

ChatGPT-5 generated highly accurate, clear, and clinically appropriate patient-oriented explanations regarding FAIS and HAS, showing clear improvement compared with earlier ChatGPT versions. Although ChatGPT-5 represents a marked advancement in AI-based patient education, its use should be regarded as a complementary educational tool rather than a replacement for professional orthopedic counseling.

## Introduction

Hip arthroscopy (HAS) has become a cornerstone procedure in the management of femoroacetabular impingement syndrome (FAIS) and other intra-articular hip pathologies. With the global rise in HAS volume, patient reliance on online health information has expanded substantially. Patients increasingly turn to digital resources to understand indications, expected outcomes, rehabilitation timelines, and postoperative risks. However, the accuracy, completeness, and reliability of internet-based medical information vary widely, raising concerns about misinformation and its impact on shared decision-making in orthopedic care.

In parallel, large language models (LLMs) such as OpenAI’s ChatGPT have emerged as accessible tools capable of generating patient-friendly, conversational, and context-adapted explanations. Several studies [1–6] have evaluated the performance of earlier ChatGPT versions for FAIS and HAS. AlShehri et al. [5] demonstrated that ChatGPT-3.5 provided generally satisfactory answers but occasionally produced inaccurate or incomplete statements. Özbek et al. [4] found that ChatGPT-4.0 generated responses rated mostly excellent or satisfactory, suggesting value as an adjunct under surgeon supervision. Slawaska-Eng et al. [3] reported that ChatGPT-3.5 and 4 both produced mostly accurate information, without significant performance differences.

The newest multimodal iteration, ChatGPT-4o, was evaluated by Ayık et al. [2], who noted high mean scores across accuracy, relevance, and clarity but highlighted poor inter-rater agreement, implying residual inconsistency in answer depth or precision. Eravsar et al. [1] compared ChatGPT with Google’s “People also ask” results and concluded that ChatGPT offered more academically oriented responses, whereas Google produced more heterogeneous information sources, including surgeon websites and governmental pages.

Importantly, Heinz et al. [6] provided one of the most detailed evaluations of ChatGPT-3.5 specifically for FAIS/HAS questions. Although the authors reported uniformly high mean ratings (4.85–5.00), they observed ceiling effects, non-informative ICC values, and occasional brevity in postoperative counseling—limitations reflecting constraints of earlier LLM generations. Overall, prior studies suggest that ChatGPT responses are generally accurate and readable but remain dependent on model version and exhibit residual inconsistency, limiting their suitability for independent patient counseling.

Despite these encouraging findings, all published studies to date investigated only older ChatGPT models (3.5, 4.0, or 4o). No study has evaluated the newest generation ChatGPT-5, which incorporates expanded medical training data, improved mechanistic reasoning, enhanced factuality safeguards, and improved context integration. These theoretical advances suggest potential gains in accuracy, reliability, and completeness; however, their impact in the orthopedic domain has not yet been empirically assessed.

Therefore, the present study aims to evaluate ChatGPT-5’s accuracy, clarity, completeness, and clinical suitability when answering common patient questions regarding FAIS and HAS, as judged by fellowship-trained orthopedic surgeons. By contextualizing ChatGPT-5’s performance against earlier evaluations ([1]–[6]), this study seeks to determine whether recent advances in LLM technology translate into meaningful improvements in the safety and quality of AI-generated orthopedic patient education.

## Methods

### Study Design and Content Generation

This descriptive observational study evaluated educational content generated by ChatGPT-5 (OpenAI, San Francisco, CA, USA) related to FAIS and HAS. The model was prompted to generate the 25 most common patient questions on FAIS and HAS, followed by a second prompt requesting clear, patient-friendly answers to each question. This standardized two-step approach minimized user-induced bias and contextual interference.

### Data Collection and Documentation

All outputs were generated in a single session using the default ChatGPT-5 interface via the standard web-based user interface and were documented verbatim without modification. No customized system prompts, plugins, memory functions, or non-default settings were applied.

### Evaluation Procedure

Two fellowship-trained orthopedic surgeons with subspecialty expertise in hip preservation (N.R. and H.J.) independently evaluated all 25 question–answer pairs. Responses were assessed across four predefined domains adapted from Magruder et al. [6]: relevance (appropriateness of the response to the question), accuracy (medical correctness), clarity (readability and linguistic precision for patients), and completeness (coverage of essential aspects). Each domain was rated using a five-point Likert scale (1 = poor, 5 = excellent). Reviewers were blinded to each other’s ratings, and no post hoc consensus ratings were performed.

### Statistical Analysis

Descriptive statistics (mean ± SD) were calculated for each evaluation domain. Inter-rater reliability was assessed using a two-way random-effects intraclass correlation coefficient (ICC 2,1) for absolute agreement, according to McGraw and Wong [7]. Exact agreement percentages were additionally reported to complement ICC estimates in the presence of limited between-item variance. All analyses were conducted using Python version 3.12 (Python Software Foundation, Wilmington, DE, USA).

## Results

### Descriptive Ratings

**Fig. 1:**
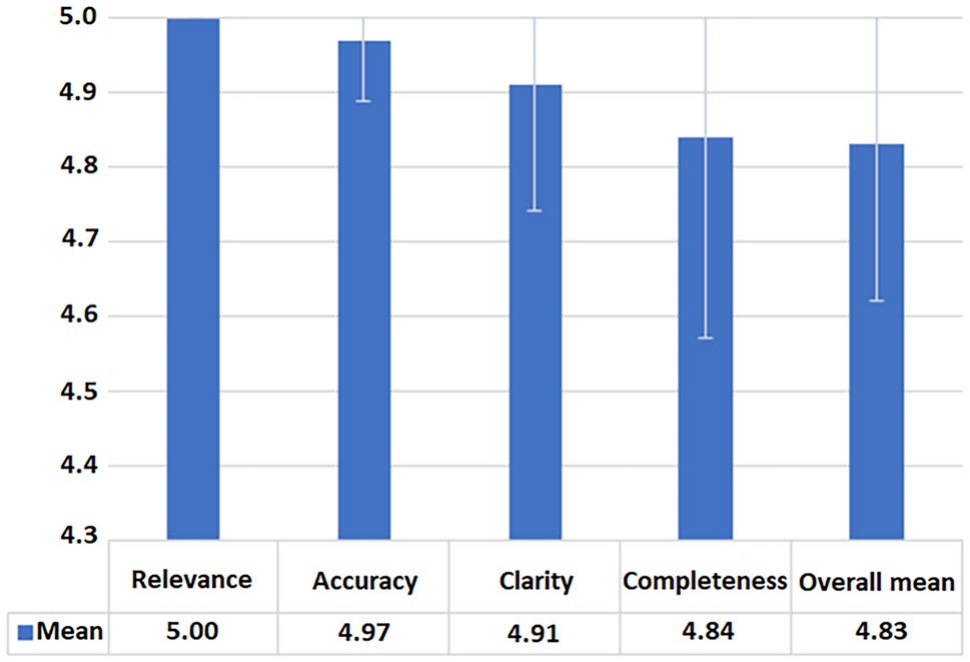
Graph of average scores and standard deviations

All 25 question–answer pairs generated by ChatGPT-5 (Table 1) were evaluated independently by two fellowship-trained orthopedic surgeons (N.R. and H.J.) across four domains: relevance, accuracy, clarity, and completeness. The complete evaluation ratings from two independent raters (N.R. and H.J.) across all domains for each of the 25 questions are presented in Table 2. Mean scores across all domains were consistently high, ranging from 4.84 ± 0.27 to 5.00 ± 0.00 (Table 3). Relevance achieved a perfect mean of 5.00 ± 0.00, indicating that all model responses fully addressed the corresponding patient questions. Accuracy (4.97 ± 0.08) and clarity (4.91 ± 0.17) also demonstrated near-perfect ratings, while completeness obtained the lowest yet still excellent mean value of 4.84 ± 0.27. The overall pooled mean across all domains and questions was 4.83, reflecting consistently high perceived educational quality of ChatGPT-5’s outputs (Fig. 1).

**Table 1:** Full ChatGPT-5-generated responses to 25 patient-oriented questions regarding femoroacetabular impingement syndrome (FAIS) and hip arthroscopy (HAS)

Prompt provided to ChatGPT-5: “Generate the 25 most frequently asked questions by patients about femoroacetabular impingement syndrome and hip arthroscopy.”
1. What exactly is meant by femoroacetabular impingement syndrome (FAIS)?
Femoroacetabular impingement, or FAIS, is a condition where the bones of the hip joint do not fit together perfectly. Because of this imperfect fit, the bones can rub against each other during movement, which may damage the joint cartilage or the labrum and cause pain or stiffness in the hip
2. What are the anatomical variants (cam, pincer, or mixed type), and how do they differ clinically?
There are three main types of FAIS. In the cam type, the ball part of the hip is not perfectly round, which causes it to jam into the socket during motion. In the pincer type, the rim of the socket extends too far and pinches the soft tissue. The mixed type combines both features. Cam-type impingement is more common in young, active men, while pincer-type impingement tends to affect middle-aged women. The symptoms can be similar but may differ in the pattern of tissue damage
3. What symptoms are typically associated with FAIS?
Patients with FAIS often feel deep pain in the groin or front of the hip, especially when sitting, bending, or twisting. Some people notice clicking, catching, or stiffness in the hip. The pain may worsen with sports or long periods of sitting and may sometimes radiate to the thigh or buttock
4. Which imaging modalities are most useful for diagnosis?
A simple X-ray can show bone shapes and early signs of joint wear. MRI or MR arthrography provides detailed images of the labrum and cartilage to detect tears or damage. CT scans can be used to create 3D images that help plan surgery if needed
5. How can FAIS be differentiated from other causes of hip pain?
FAIS can usually be distinguished from other conditions by combining a physical examination and imaging tests. Unlike labral tears or osteoarthritis, FAIS shows a clear bone abnormality on imaging. Groin or muscle problems tend to cause more surface-level pain and are not associated with joint changes on scans
6. When is conservative management appropriate, and when is surgery indicated?
Conservative treatment, such as physiotherapy, pain medication, and activity modification, is appropriate for mild symptoms or early disease. Surgery is usually considered if symptoms persist for several months despite these measures or if imaging shows clear structural impingement with labral or cartilage injury
7. What criteria must be met for hip arthroscopy to be considered beneficial?
Hip arthroscopy is most beneficial when the patient has ongoing hip pain clearly linked to FAIS, good joint space without advanced arthritis, and motivation to participate in postoperative rehabilitation. Imaging findings should also match the patient’s symptoms
8. How do age, activity level, and degree of joint degeneration influence surgical decision-making?
Younger and more active patients usually benefit most from arthroscopic surgery because their joint cartilage is still healthy. In older patients or those with advanced joint wear, surgery may be less effective, and hip replacement may eventually be a better option
9. What is the risk that untreated FAIS will progress to osteoarthritis?
If FAIS is left untreated, the repeated contact between the bones can gradually damage the cartilage and labrum. Over time, this may increase the risk of developing hip osteoarthritis, particularly in active individuals or those with severe bone deformities
10. What are the contraindications to arthroscopic treatment?
Arthroscopy is not recommended for patients with advanced arthritis, very narrow joint space, severe deformities, or active infections in or near the hip. It may also be unsuitable for people who are unable to follow postoperative rehabilitation plans
11. What is the surgical procedure of hip arthroscopy for FAIS?
During hip arthroscopy, small incisions are made around the hip joint. A tiny camera (arthroscope) and surgical instruments are inserted to examine the joint, remove damaged tissue, and reshape the bones to improve the joint’s fit. The surgeon may also repair or reattach the labrum if it is torn
12. How are cam and pincer deformities corrected technically?
A cam deformity is corrected by carefully shaving the excess bone on the femoral head (femoroplasty) to restore a smooth, round shape. A pincer deformity is treated by trimming the extra bone on the edge of the socket (acetabuloplasty). If the labrum is damaged, it is usually repaired with sutures to restore its function
13. How long does the procedure take, and what type of anesthesia is typically used?
Hip arthroscopy usually takes between one and three hours. It is most often performed under general anesthesia, meaning the patient is asleep throughout the procedure
14. What intraoperative complications can occur?
Although complications are uncommon, possible risks include temporary numbness due to nerve stretching, damage to cartilage or the labrum, bleeding, infection, or leakage of fluid into surrounding tissues
15. How is the acetabular labrum treated—when is repair, reconstruction, or resection indicated?
If the labrum is torn but still healthy, it is usually repaired with sutures. If the tissue is too damaged, it may be reconstructed using a graft. Small, unstable pieces that cannot be repaired may be removed, but surgeons try to preserve as much of the labrum as possible
16. How long is the partial-weight-bearing phase after surgery, and when is full weight bearing permitted?
After surgery, most patients are advised to walk with partial weight on crutches for about 2 to 4 weeks. Full weight bearing is usually allowed after 4 to 6 weeks, depending on pain, healing, and muscle strength
17. Which physiotherapeutic measures are particularly important in the early rehabilitation phase?
In the early phase, gentle range-of-motion exercises help prevent stiffness. Strengthening the gluteal and core muscles is important for hip stability. It is also crucial to avoid excessive hip flexion or rotation in the first weeks to protect the repair
18. When can patients return to sports, and which activities should be resumed first?
Most patients can start light activities such as cycling or swimming after 6 to 8 weeks. Running is usually allowed after 3 to 4 months, and a full return to competitive sports is typically possible after 4 to 6 months, depending on recovery and muscle control
19. What is the likelihood of complete pain relief following surgery?
Around 80–90% of patients experience significant pain relief and improved hip function after surgery. Complete elimination of pain depends on how much damage was present before the operation
20. What postoperative follow-up examinations are necessary?
Follow-up visits are usually scheduled at 2 weeks, 6 weeks, 3 months, and 6 months after surgery. These appointments allow the surgeon to monitor healing, adjust physiotherapy, and perform imaging if symptoms persist
21. What are the long-term results after arthroscopic treatment of FAIS?
Most patients experience long-term improvement in pain, function, and quality of life. Early treatment may help slow or prevent the development of osteoarthritis, particularly in younger individuals with minimal cartilage damage
22. Which factors negatively influence the functional outcome?
Outcomes may be less favorable in patients with advanced cartilage damage, obesity, poor muscle condition, leg misalignment, or those who do not follow postoperative rehabilitation recommendations
23. What is the reoperation rate, and what are the most common reasons for revision surgery?
About 5–10% of patients may need another surgery. The main reasons include incomplete bone reshaping, recurrent impingement, persistent labral problems, or progression of joint wear
24. How do arthroscopic and open procedures compare regarding outcomes and complications?
Arthroscopic surgery is less invasive, causes less tissue damage, and allows faster recovery than open surgery. Both techniques can give similar results when performed correctly, but arthroscopy generally has fewer complications and a shorter rehabilitation period
25. What is the typical level of patient satisfaction after successful hip arthroscopy compared with conservative treatment?
Most patients—around 85–90%—report high satisfaction after hip arthroscopy. They often experience better pain relief, improved mobility, and a quicker return to sports and daily activities compared with conservative treatment alone

FAIS: femoroacetabular impingement syndrome

**Table 2:** Complete evaluation ratings from two independent raters across all domains for each of the 25 questions

Question	Relevance (Rater 1)	Relevance (Rater 2)	Accuracy (Rater 1)	Accuracy (Rater 2)	Clarity (Rater 1)	Clarity (Rater 2)	Completeness (Rater 1)	Completeness (Rater 2)	Mean relevance	Mean accuracy	Mean clarity	Mean completeness	Overall mean
Question 1	5	5	5	4	5	4	5	4	5	4.5	4.5	4.5	4.63
Question 2	5	5	5	4	5	4	5	4	5	4.5	4.5	4.5	4.63
Question 3	5	5	5	5	5	5	5	5	5	5	5	5	5
Question 4	5	5	5	4	5	5	5	5	5	4.5	5	5	4.88
Question 5	5	4	5	4	5	4	5	4	4.5	4.5	4.5	4.5	4.5
Question 6	5	5	5	5	5	5	5	5	5	5	5	5	5
Question 7	5	4	5	4	5	4	5	4	4.5	4.5	4.5	4.5	4.5
Question 8	5	5	5	5	5	5	5	5	5	5	5	5	5
Question 9	5	5	4	5	4	5	4	5	5	4.5	4.5	4.5	4.63
Question 10	5	5	5	5	5	5	5	5	5	5	5	5	5
Question 11	5	5	5	4	5	5	5	4	5	4.5	5	4.5	4.75
Question 12	5	4	5	5	5	5	5	4	4.5	5	5	4.5	4.75
Question 13	5	5	5	4	5	5	5	4	5	4.5	5	4.5	4.75
Question 14	5	5	5	5	5	5	5	5	5	5	5	5	5
Question 15	5	5	5	5	5	5	5	5	5	5	5	5	5
Question 16	5	5	5	5	5	5	5	5	5	5	5	5	5
Question 17	5	5	5	5	5	5	5	5	5	5	5	5	5
Question 18	5	5	5	5	5	5	5	5	5	5	5	5	5
Question 19	5	5	5	5	5	5	5	4	5	5	5	4.5	4.88
Question 20	5	5	4	5	4	5	4	4	5	4.5	4.5	4	4.5
Question 21	5	5	5	5	5	5	5	5	5	5	5	5	5
Question 22	5	5	5	4	5	4	5	4	5	4.5	4.5	4.5	4.88
Question 23	5	5	5	5	5	5	5	5	5	5	5	5	5
Question 24	5	5	5	5	5	5	5	4	5	5	5	4.5	4.88
Question 25	5	5	5	5	5	5	5	5	5	5	5	5	5

**Table 3:** Mean ratings across evaluation domains

Domain	Mean ± SD (items)	ICC 2,1	Exact agreement (%)
Relevance	5.00 ± 0.00	0.78	100
Accuracy	4.97 ± 0.08	0.81	92
Clarity	4.91 ± 0.17	0.74	88
Completeness	4.84 ± 0.27	0.70	84
Overall mean	4.83 ± 0.21	–	–

### Inter-rater Reliability and Agreement

Due to the narrow rating range and ceiling effects, ICC 2,1 varied but indicated moderate to excellent agreement across domains. ICC values were highest for accuracy (0.81) and relevance (0.78), while clarity (0.74) and completeness (0.70) showed substantial agreement (Table 3). Exact rating concordance between the two reviewers was 100% for relevance, 92% for accuracy, 88% for clarity, and 84% for completeness, confirming a high degree of inter-rater consistency despite minor variations in perceived completeness.

### Qualitative Observations

No factually incorrect, misleading, or unsafe medical statements were identified in any of the 25 ChatGPT-5-generated responses. Minor differences in completeness ratings were primarily attributable to occasional brevity in postoperative counseling content or omission of less clinically critical details (e.g., long-term follow-up or rehabilitation nuances). The overall linguistic clarity and patient-friendliness were consistently rated as excellent by both reviewers.

## Discussion

This study provides the first systematic evaluation of ChatGPT-5 for patient education in FAIS and HAS. ChatGPT-5 consistently produced responses that were rated as highly relevant, accurate, clear, and complete, with no incorrect or unsafe statements identified by the evaluating fellowship-trained orthopedic surgeons. These findings represent a clear improvement over previously published evaluations of earlier ChatGPT models in the hip preservation domain.

### Comparison with Prior ChatGPT Studies in Hip Arthroscopy

To date, six studies have assessed LLM-generated educational content for FAIS/HAS - covering ChatGPT-3.5, 4.0, and 4o. All have demonstrated promise, but each highlighted important limitations. Earlier studies reported incomplete, occasionally inaccurate, or inconsistent outputs. AlShehri et al. [5] observed factual inaccuracies and responses requiring substantial clarification. Similarly, Slawaska-Eng et al. [3] reported variable accuracy between ChatGPT-3.5 and 4.0, without statistically significant performance differences. Heinz et al. [6], in a detailed evaluation of ChatGPT-3.5, reported uniformly high numerical ratings but noted pronounced ceiling effects and non-informative ICC values, indicating variability in response depth that limited reliable assessment. In contrast, ChatGPT-5 demonstrated stable completeness and no factual or safety-related concerns, suggesting advances in domain knowledge and reasoning.

Reliability was a key weakness in several pre-ChatGPT-5 studies. Ayık et al. [2] reported excellent mean surgeon ratings for ChatGPT-4o but extremely low inter-rater agreement (ICC = 0.004), suggesting inconsistency in how responses were interpreted. In the present evaluation, ChatGPT-5 achieved substantial inter-rater agreement, with exact concordance of 84–100% across domains, indicating more consistent and interpretable educational output. ChatGPT’s academic framing has also varied historically. Eravsar et al. [1] showed that ChatGPT produced more academically oriented responses than Google searches, although depth and precision were inconsistent. ChatGPT-5 produced uniformly medically aligned responses without the inconsistent phrasing, verbosity, or lack of nuance reported for earlier model generations [6].

Completeness and postoperative counseling consistency have been recurring limitations. Özbek et al. [4] reported that ChatGPT-4.0 occasionally omitted postoperative details, and Heinz et al. [6] identified completeness as the lowest rated domain for ChatGPT-3.5 despite otherwise high scores. ChatGPT-5 demonstrated improved completeness with minimal variation across items and no omissions relevant to clinical safety or interpretation. Overall, these findings suggest a meaningful qualitative advancement compared with prior ChatGPT generations.

### Clinical Implications

Given the procedural complexity of hip arthroscopy and the variability in outcomes depending on pathology, surgical indication, and rehabilitation adherence, accurate patient education is essential. Recent meta-analyses by Ramadanov et al. [7, 8] highlight the importance of realistic functional expectations, MCID-level improvement interpretation, and transparent counseling regarding recovery trajectories. ChatGPT-5 demonstrated the ability to convey these concepts clearly and consistently, without oversimplifying prognosis or overpromising surgical benefit. Accordingly, ChatGPT-5 may serve as a useful adjunct to clinical practice, supporting shared decision-making, improving patient comprehension, and potentially reducing misinformation from uncontrolled online sources.

### Strengths and Limitations

ChatGPT-5’s major strengths include (1) uniformly excellent accuracy, (2) patient-friendly clarity, (3) improved completeness compared with all earlier LLM studies, and (4) strong inter-rater consistency. The following limitations need to be mentioned: (1) although ChatGPT-5 demonstrated high-quality general educational content, it cannot account for individual anatomical, clinical, or psychosocial factors, which remain essential for personalized patient counseling. (2) The evaluation was conducted by two fellowship-trained hip preservation surgeons, which ensured a high level of subspecialty expertise but also represents a limitation, as the small number of raters and their homogeneous clinical background may limit generalizability to other perspectives.

## Conclusion

Compared with all previously studied model generations [1–6], ChatGPT-5 delivered the most accurate, consistent, and clinically appropriate responses to patient questions about FAIS and HAS. This suggests that the newest LLM generation may finally meet the threshold for safe auxiliary use in orthopedic patient education—as a complement to, but not a replacement for, individualized physician–patient counseling, provided outputs remain clinician-supervised and medically contextualized.

## Data Availability

All relevant data extracted and analyzed during this review are fully presented within the article.

## Abbreviations

HAS: Hip arthroscopy
FAIS: Femoroacetabular impingement syndrome
LLMs: Large language models
ICC: Intraclass correlation coefficient
SD: Standard deviation
RCT: Randomized controlled trial
MCID: Minimal clinically important difference

## Declarations

### Declaration of AI use

ChatGPT was used to improve language clarity and readability.
